# Supplementary material for: Neonatal apnea and hypopnea prediction in infants with Robin sequence with neural additive models for time series
Source: PLOS Digit Health. 2024 Dec 13;3(12):e0000678. doi: 10.1371/journal.pdig.0000678 (PMC11642933; doi:10.1371/journal.pdig.0000678)
Supplement: S1 Fig — (PDF) [file pdig.0000678.s001.pdf]

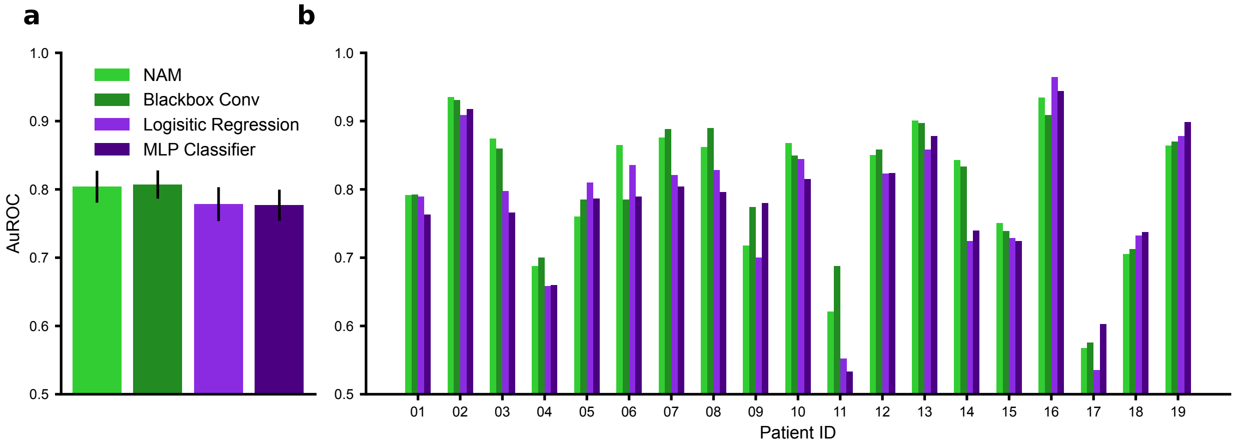

S1 Figure. Comparison of neural additive model (NAM) to baselines models. a) Average AuROC over all 19 infants of the neural additive model compared to the blackbox neural network as well as the feature-based logistic regression and multi-layer perceptron classifier. Error bars indicate standard error of the mean over the 19 infants. **b)** Performance of the four models for the 19 individual infants.
