# Supplementary material for: Neonatal apnea and hypopnea prediction in infants with Robin sequence with neural additive models for time series
Source: PLOS Digit Health. 2024 Dec 13;3(12):e0000678. doi: 10.1371/journal.pdig.0000678 (PMC11642933; doi:10.1371/journal.pdig.0000678)
Supplement: S1 Appendix — (PDF) [file pdig.0000678.s002.pdf]

Appendix

Details on the significance analysis

For a rigorous performance comparison between the Neural Additive Model (NAM) and single modality networks, we ran the complete training and testing pipeline ten times and compute the average AuROC and standard deviation over all patients. We then performed a significance analysis over the differences in average performance between the NAM and the single modality networks as well as the baseline models. To this end, we computed Wilcoxon signed-rank tests over the $n = 19$ patients. We further extended this significance analysis by comparing all pairs of single-modality network performances, again using Wilcoxon signed-rank tests. We also performed permutation permutation tests [1] for all patients for both the NAM and all single modality networks. See tables in supporting information for all results.

Details on baselines and feature engineering

Feature-based baselines To compare our NAM with more classical approaches, we trained a logistic regression and a multi-layer perceptron (MLP) classifier using the same leave-one-out approach. To make these baselines competitive, we computed a total of 24 features across the six signal modalities. For heart rate, SpO_2, and PCO_2, the average and range (i.e., maximum minus minimum) were computed over each 30-second time window to capture decelerations or abnormally low or high values. For the oscillatory modalities, that is, the nasal pressure, the thoracic and abdominal respiratory efforts, and the pulse plethysmogram, we computed (higher-order) moments in time and spectral domain to capture differences in the "regularity" of the oscillatory dynamics. More specifically, in the time domain, we computed the third and fourth central moment (skewness and kurtosis). In the spectral domain, we computed moments based on the spectral centroid (SC). The SC is given by

$$\mathrm{SC} = \frac{\sum_{n=0}^{N-1} f(n) \cdot X(n)}{\sum_{n=0}^{N-1} X(n)},$$

where $f(n)$ represents the frequency at bin n, $X(n)$ represents the magnitude (or power) of the signal at frequency bin n, and N is the total number of frequency bins. Based on the spectral centroid, we also computed the spectral spread, skewness and kurtosis (see [2] for details).

Blackbox neural network To compare our NAM to an uninterpretable but powerful model, we employed the individual single-modality networks used in the NAM to extract features into a high-dimensional latent space (20 dimensions per modality and 120 dimensions in total). This high-dimensional latent embedding is then passed through an MLP to perform classification. We trained both the single modality networks and the MLP end-to-end in a single architecture. This architecture is more powerful and expressive than the NAM, but the ability to compute additive contributions or visualize activations per modality is lost.

Training of baseline models We trained all baseline models using the identical leave-one-out approach as described for the NAM. Hyperparameters were chosen independently for each baseline model. For the MLP classifier and blackbox neural network, we again used the Adam optimizer [3, 4] with a learning rate of 0.0001 and a weight decay of 0.01, and trained for 10 epochs. For the logistic regression, we used the L-BFGS optimizer [5] with a learning rate of 0.01, a history size of 10, a L_2-regularization strength of 0.001 and trained for 100 iterations.

Different time window lengths To investigate whether longer prediction windows further improve prediction, we conducted an experiment with 60-second time windows. To ensure a fair comparison with 30-second time windows, we matched 30 and 60-second time windows one-to-one. This was achieved by first extracting 60-second time windows and then removing the second half to obtain the matching 30-second time windows. As a result, the 30-second time windows used in this experiment differ from those used in the main text.

We then trained the NAM on both time window lengths using the same hyperparameters as in the main experiment. The NAM with 60-second time windows achieved an average AuROC of 0.762, and the NAM with

30-second time windows achieved an AuROC of 0.764. Thus, there was no improvement with increasing time window length. This result is consistent with our earlier experiment, which showed that prediction performance deteriorates significantly with increasing prediction horizon. Clinically, the occurrence of irregularities in the polysomnography recording is expected to occur close to the onset of an apnea or hypopnea.

Description and definition of adverse events

The criteria used to annotate the recorded polygraphy signals are given in the following. More details can be found in [6].

1. Obstructive apnea
   - Lasts for ≥ 2 breaths with respiratory effort present on inductance belt
2. Central apnea
   - Lasts for ≥ 2 breaths with associated SpO_2 decrease by $\geq 3\%$
   - Lasts for ≥ 20 seconds
   - Arousal *or* heart rate < 50 bpm for ≥ 5 seconds *or* heart rate < 60 bpm for > 15 seconds
3. Mixed apnea
   - Lasts for ≥ 2 breaths with component of both obstructive and central apnea
4. Obstructive hypopnea
   - Nasal flow $\leq 70\%$ of baseline for ≥ 2 breaths with associated SpO_2 decrease by $\geq 3\%$
   - Arousal
   - Hypopnea with increased inspiratory flattening
   - Thoracoabdominal paradox
5. Central hypopnea
   - Without above-mentioned features of obstruction
6. Hypoxia
   - SpO_2 decrease by $\geq 3\%$ within a 5 seconds duration
7. Movement
   - Gross movement observed for at least 15 seconds
   - Eye opening
   - Two episodes of movement need to be separated by at least 15 seconds

Data and code availability

The dataset analyzed in this study is available on Zenodo: `https://zenodo.org/record/7711137`. The code for all experiments is available at `https://github.com/mackelab/neonatal_apnea_prediction`.

References

1. Ojala M, Garriga GC. Permutation Tests for Studying Classifier Performance. Journal of Machine Learning Research. 2010;11(6):1833–1863.

2. Peeters G, Giordano BL, Susini P, Misdariis N, McAdams S. The timbre toolbox: Extracting audio descriptors from musical signals. The Journal of the Acoustical Society of America. 2011;130(5):2902–2916.

3. Kingma DP, Ba J. Adam: A method for stochastic optimization. In: arXiv preprint arXiv:1412.6980; 2014.

4. Loshchilov I, Hutter F, et al. Fixing weight decay regularization in Adam. arXiv preprint arXiv:171105101. 2017;5.

5. Liu DC, Nocedal J. On the limited memory BFGS method for large scale optimization. Mathematical programming. 1989;45(1):503–528.

6. Lim K, Quante M, Dijkstra T, Hilbert-Moessner G, Wiechers C, Dargaville P, et al. Should obstructive hypopneas be included when analyzing sleep studies in infants with Robin Sequence? Sleep Medicine. 2022;98:9–12.
