# Supplementary material for: Neonatal apnea and hypopnea prediction in infants with Robin sequence with neural additive models for time series
Source: PLOS Digit Health. 2024 Dec 13;3(12):e0000678. doi: 10.1371/journal.pdig.0000678 (PMC11642933; doi:10.1371/journal.pdig.0000678)
Supplement: S1 Table — (PDF) [file pdig.0000678.s003.pdf]

S1 Table. NAM vs. single modality networks vs. baselines. Average AuROC and standard deviation across different runs (Seed-SD) of the neural additive model (NAM), signal modality networks (with NP for nasal pressure, T+A for thoracic and abdominal respiratory effort, HR for heart rate, PPG for photoplethysmogram, and SpO_2 and PCO_2 levels), and baseline models (blackbox neural network, logistic regression, multi-layer perceptron (MLP) classifier) over 10 independent training and testing runs. The Wilcoxon tests are computed over the $n = 19$ patients with the AuROC performances for each patient averaged over all 10 runs. All tests are two-sided.

Model	AuROC	Seed-SD	Wilcoxon
NAM	0.803	0.0077	-
NP	0.753	0.0067	$= 0.009$
T+A	0.735	0.0042	< 0.001
SpO_2	0.733	0.0058	$= 0.006$
PPG	0.694	0.0074	< 0.001
HR	0.627	0.0073	< 0.001
PCO_2	0.614	0.0077	< 0.001
Blackbox	0.807	0.0078	$= 0.623$
Log. Reg.	0.778	0.0016	$= 0.014$
MLP	0.777	0.0027	$= 0.072$
Reduced NAM	0.802	0.0045	$= 0.859$
