# Supplementary material for: Neonatal apnea and hypopnea prediction in infants with Robin sequence with neural additive models for time series
Source: PLOS Digit Health. 2024 Dec 13;3(12):e0000678. doi: 10.1371/journal.pdig.0000678 (PMC11642933; doi:10.1371/journal.pdig.0000678)
Supplement: S2 Table — (PDF) [file pdig.0000678.s004.pdf]

S2 Table. Statistical test results for single modality network performances. The Wilcoxon tests are computed over the $n = 19$ patients where the AuROC performance for each patient is the average over all 10 runs. All tests are two-sided. NP for nasal pressure, T+A for thoracic and abdominal respiratory effort, HR for heart rate, PPG for photoplethysmogram, and SpO_2 and PCO_2 levels.

Model	NP	T+A	SpO_2,	PPG	HR
T+A	$= 0.032$				
SpO_2	$= 0.241$	$= 0.829$			
PPG	$= 0.011$	$= 0.087$	$= 0.568$		
HR	$= 0.007$	$= 0.016$	$= 0.005$	$= 0.169$	
PCO_2	$= 0.012$	$= 0.018$	$= 0.003$	$= 0.123$	$= 0.49$
