# Supplementary material for: Neonatal apnea and hypopnea prediction in infants with Robin sequence with neural additive models for time series
Source: PLOS Digit Health. 2024 Dec 13;3(12):e0000678. doi: 10.1371/journal.pdig.0000678 (PMC11642933; doi:10.1371/journal.pdig.0000678)
Supplement: S3 Table — (PDF) [file pdig.0000678.s005.pdf]

S3 Table. Permutation tests for both the NAM and all single modality networks. The permutation tests were performed with 1024 permutations. Exact p-values are given for values larger than 0.001. NP for nasal pressure, T+A for thoracic and abdominal respiratory effort, HR for heart rate, PPG for photoplethysmogram, and SpO_2 and PCO_2 levels.

ID	NAM	NP	T+A	SpO_2	PPG	HR	PCO_2
01	0.001	0.001	0.001	0.001	0.001	0.001	0.017
02	0.001	0.001	0.001	0.001	0.001	0.001	0.001
03	0.001	0.001	0.002	0.001	0.001	0.001	0.001
04	0.001	0.001	0.005	0.062	0.008	0.002	0.001
05	0.001	0.001	0.001	0.001	0.008	0.374	0.983
06	0.001	0.001	0.001	0.001	0.001	0.607	0.001
07	0.001	0.001	0.001	0.001	0.001	0.044	0.921
08	0.001	0.001	0.001	0.001	0.001	0.037	0.998
09	0.001	0.03	0.02	0.001	0.2	0.027	0.005
10	0.001	0.001	0.001	0.001	0.001	0.997	0.7
11	0.004	0.845	0.001	0.001	0.727	0.001	0.003
12	0.001	0.001	0.001	0.001	0.008	0.001	0.001
13	0.001	0.001	0.001	0.001	0.001	0.001	0.001
14	0.001	0.001	0.001	0.001	0.001	0.001	0.07
15	0.001	0.001	0.001	0.006	0.008	0.016	0.006
16	0.001	0.001	0.001	0.001	0.001	0.366	0.001
17	0.121	0.157	0.558	0.713	0.103	0.031	0.004
18	0.001	0.001	0.001	0.001	0.001	0.853	0.778
19	0.001	0.001	0.014	0.001	0.661	0.001	0.001
