# Supplementary material for: Neonatal apnea and hypopnea prediction in infants with Robin sequence with neural additive models for time series
Source: PLOS Digit Health. 2024 Dec 13;3(12):e0000678. doi: 10.1371/journal.pdig.0000678 (PMC11642933; doi:10.1371/journal.pdig.0000678)
Supplement: S4 Table — (PDF) [file pdig.0000678.s006.pdf]

S4 Table. Confusion matrices for individual patients. Decision threshold set to achieve 20% false positive rate (FPR). TN for true negatives, FP for false positives, FN for false negatives, and TP for true positives.

ID	TN	FP	FN	TP
01	123	30	58	66
02	122	22	9	61
03	62	15	10	35
04	215	54	36	29
05	24	5	26	31
06	197	50	15	55
07	137	32	21	73
08	109	26	15	60
09	45	10	34	36
10	149	36	13	43
11	33	7	19	16
12	81	20	15	62
13	170	43	10	49
14	278	71	20	46
15	424	97	14	14
16	44	10	23	43
17	468	114	14	10
18	111	26	34	27
19	46	8	7	31
