# Supplementary material for: Neonatal apnea and hypopnea prediction in infants with Robin sequence with neural additive models for time series
Source: PLOS Digit Health. 2024 Dec 13;3(12):e0000678. doi: 10.1371/journal.pdig.0000678 (PMC11642933; doi:10.1371/journal.pdig.0000678)
Supplement: Attachment — Submitted filename: response_letter_neonatal_apnea_prediction.pdf [file pdig.0000678.s007.pdf]

Response letter

We thank the reviewers and the editor for their positive assessment and constructive suggestions. Based on these comments, we have performed additional experiments comparing our model to more classical baselines, i.e. logistic regression and a multi-layer perceptron classifier based on 24 features. In addition, we now compare our NAM to a "blackbox" convolutional neural network to quantify any trade-off between interpretability and performance, as suggested by Reviewer 2. In short, we find that our NAM performs slightly better than the classical feature-based baselines and on par with the blackbox neural network. We have also addressed questions and comments from both reviewers, and have significantly expanded the Discussion and Limitations section. We provide detailed responses to each reviewer below, and have revised the manuscript accordingly, with additional clarifications, discussions and supplementary figures and tables. The original reviewer comments are shown below in blue, our responses in black, and the corresponding changes to the manuscript in red.

Reviewer #1:

Major comments: The methodology is not transparent, and the analysis rationale needs more explanations.

1. Descriptive statistics for 19 patients in this study are missing. How many of them encountered Neonatal apnea and hypopnea? Is the prevalence similar to that of this disease population?

We provide descriptive statistics in the first subsection of the Results section entitled “Descriptive statistics”. All 19 infants were diagnosed with Pierre-Robin sequence and experienced apneas and hypopneas during the whole-night recording. The prevalence (taken to indicate the number of apnea and hypopnea events per hour) is similar to what is known of this group of patients (Lim et al. 2022, Buchenau et al. 2017).

“Should obstructive hypopneas be included when analyzing sleep studies in infants with Robin Sequence?”, Lim et al., Sleep Medicine, 2022

“Functional treatment of airway obstruction and feeding problems in infants with Robin sequence”, Buchenau et al., Arch Dis Child Fetal Neonatal, 2017

2. Page 5: “To discriminate between target and control time windows, a neural additive model (NAM) [1] was trained and tested with patient-based leave-one-out cross validation” Is time series suitable for such cross-validation design that ignore the temporal pattern?

Our model does not ignore temporal patterns as it directly operates on 30-second time windows of the underlying polysomnographic recording. To ensure that there is no leakage of information between training and test data set, we evaluated the performance of our model on time windows from a completely held-out infant (and repeated this for every infant in the dataset). As such, this leave-one-out approach is especially suitable and conservative in our time series setting, as patients are independent and no leakage of information can occur. We now added the following sentence to the Methods section to further clarify this point (line 102):

This leave-one-out approach ensured that there was no information leakage between the training and test sets, as the time windows on which the model was tested were extracted from a completely independent patient.

3. Six signals were used for the analysis: Nasal pressure (NP), the sum of thoracic and abdominal respiratory effort (T+A), photoplethysmogram(PPG), heart rate (HR), SpO2, and PCO2 levels. Is there any redundant signal for prediction? Could the model be reduced to fewer signals without sacrificing the predictive ability?

We thank the reviewer for this interesting suggestion. We have now trained a NAM using only the three most informative modalities (according to our previous analysis with the full NAM and the single modality networks). These were the nasal pressure, the thoracic and abdominal respiratory effort, and the SpO_2 level.
The NAM using only these three modalities achieved an average AuROC of 0.801, which is not significantly different from the NAM using all six signal modalities. However, as we have shown in Table A1, the NAM was significantly better than any single-modality network. We have included these results in the paper (line 265):

Based on this modality importance analysis, we trained a NAM using only the three most predictive signal modalities, that is, the nasal pressure, the sum of thoracic and abdominal respiratory effort, and the SpO_2 level. The “reduced” NAM achieved an average AuROC of 0.802 that was not significantly different from the full NAM using all six modalities (Table A1). As discussed in the comparison with the blackbox model, this result suggests some redundancy in the predictive information between modalities.

4. The training and testing procedure is not clear. How did the author separate the dataset?

We extracted the 30-second time windows from all 19 patients. Then, out of the 19 patients, one was selected for testing, and the remaining 18 were used for training and validation. This nested cross-validation is a conservative approach that guarantees that there is no leakage of information between the set of time windows used for training/validation and testing, as patients can be assumed to be totally independent. We

now more clearly point this out in the paper in line 99 and line 102 (see response to point 2).

5. The confusion matrix is missing. It is unclear how the classification results perform.

We report several metrics to show the classification performance, including the threshold-independent area under the receiver operating characteristic (AuROC), the true positive rate (TPR) and precision at different fixed levels of false positive rate (FPR). Additionally, we have now added Table A4 to the appendix containing the confusion matrices per infant for a decision threshold based on a 20% FPR.

6. CNN could analyze signals but may not be optimal compared to other equations or models.

We agree that other (and potentially less interpretable) models should be compared to our NAM approach. We have added two more "classical" baselines based on feature engineering, i.e., logistic regression and a multi-layer-perceptron classifier based on 24 features (see line 166 and line 219 in the manuscript, and the Appendix, or our response to the second reviewer for more details). While both of these approaches performed well, the NAM still achieved a higher average AuROC.

7. Line 198: To assess the significance of potential differences between modalities, we repeated our training procedure five times and computed Wilcoxon signed-rank (pwil) tests over the n = 19 patients. However, the appendix says r=10. Moreover, how did the authors compare the five repeats? What is the two-sample t-test for? What are the authors comparing? Are these values independent?

We performed two types of tests to assert the differences between the NAM, different single-modality networks, and baseline models. The important test is the Wilcoxon signed-rank test to establish that the NAM performed significantly better across patients. It was based on the average performance values for each infant averaged over ten runs (not five, we have corrected this) with different random seeds.

Additionally, to show that the performance differences are stable over 10 different repeats, we initially used t-tests to compare the distribution of performances from different seeds averaged over all patients.

However, we agree with the reviewer that the additional t-tests over the random seeds are less important and can lead to confusion. We therefore decided to remove them from the manuscript. We now only report the standard deviations across the repeats (see Table A1) and write (line 180):

To assess the significance of potential differences between different models, we always repeated our training procedure ten times to obtain average performances for each infant and computed Wilcoxon signed-rank tests over the n=19 patients. Furthermore, we performed permutation tests to check whether models perform significantly better than random. See Appendix for more details on the statistical tests.

8. Tables 2, 3, and 4 seem to present similar research questions. However, it is not explained in the text, and I do not understand the meaning of these tests.

We apologize for not making the tests we performed more clear and for not referencing them in the main text. Table 2 (now Table A1) shows that the NAM performed significantly better than any single modality network. To provide additional information, Table 3 (now Table A2) computes p-values for all remaining pairs within the single modality performances. Table 4 did the same for the distribution of additive contributions within the NAM. We agree with the reviewer that Tables 3 and 4 were similar and therefore decided to remove Table 4 from the Appendix. Furthermore, we made sure to reference the remaining tables in the main text.

9. Does Table 5 mean that single-modality networks are good enough?

Table 5 (now Table A4) presents the results of our permutation tests and therefore shows that the NAM, as well as the single modality networks, perform significantly better than random for most patients.

However, the best performing single modality network (using the nasal pressure signal) was significantly worse than the neural additive model (Table A1).
As already acknowledged in the manuscript, in further studies, the nasal pressure might be used as the single predictor for neonatal apnea and hypopnea in infants with Pierre-Robin sequence, especially when clinicians want to reduce the strain on the infant.
Alternatively, as suggested by the reviewer, a subset of the six modalities might suffice (e.g. the three most predictive modalities as in the experiment above).
We agree with the reviewer that these are relevant questions that deserve investigation in follow-up work, where such a model is applied in a real-time setting.

10. Does the performance of this research meet the criteria for clinical use? The TPR and precision are not satisfying (Table 1).

Our study represents a step towards automated prediction (and potentially intervention) of neonatal apneas and hypopneas. Further studies are needed to provide clear evidence for or against the applicability of our results in clinical practice. We clearly acknowledge

this in our Limitations section and have further expanded the relevant parts. See line 327, line 336 and line 343:

A warning system that produces many false negatives would not significantly reduce the stress an infant experiences from repeated apneas and hypopneas. Conversely, a warning system that produces many false positives would potentially place additional stress on the infant by prompting unnecessary interventions.

We also excluded any periods of infant movement from analysis that would need to be dealt with in a real-world application.
As such, this study represents a first step toward automated systems, but the practical validity of our model requires further study. Because our used dataset consisted of only 19 infants with Robin sequence who have a homogeneous pathophysiology, future studies need to apply the NAM to larger datasets and datasets collected in other settings with different patient demographics and more heterogeneous pathophysiology to provide external validation of our approach.

Finally, while the use of such automated machine learning systems in neonatal intensive care and health care in general is promising, the use of such systems raises challenging ethical considerations regarding (parental) consent, privacy, and accountability for potential harm.

Nevertheless, we believe that our results are a promising first step towards future automated systems, as our model is able to predict individual neonatal apneas and hypopneas significantly better than chance.

11. Correct Positive and Correct Negative in the manuscript are generally referred to as True Positive and True Negative.

Thank you for pointing this out. We have changed it in the manuscript figure.

Reviewer #2:

The study by Vetter et al. investigates the prediction of neonatal apnea and hypopnea in infants with Robin sequence using neural additive models (NAMs) for time series analysis. This research utilizes machine learning techniques to enhance predictive accuracy and facilitate early interventions for at-risk neonates. The manuscript is well-written with clear sections and logical flow. The technical language seems appropriate for the target audience of medical and AI researchers.

The study is methodologically sound and offers promising results that could improve clinical outcomes, which make it clinically relevant. By addressing the suggested manuscript improvements (see below), the manuscript has the potential to make an important contribution to the field of neonatology.

Major Points
1. Relevance and Originality:
Relevance: The study addresses a critical aspect of neonatal care, focusing on predicting apnea and hypopnea in infants with Robin sequence, which significantly impacts neonatal morbidity and mortality.
Originality: The application of NAMs for time series in this context is novel, providing a new approach compared to traditional predictive models.

2. Methodology:
Data Collection: The manuscript provides detailed information on data collection from a single center NICU (Tubingen University Hospital) with a focus on infants diagnosed with Robin sequence. The inclusion criteria and data sampling methods are well-described.
Model Development: The architecture of the NAM is clearly explained. However, a comparative analysis with traditional models, such as logistic regression, support vector machines, or simpler neural networks, would be beneficial to further differentiate why NAMs are favorable in this context.

Validation: Cross-validation techniques ensure the model's robustness. However, external validation on a separate cohort would be ideal to strengthen the findings. If this is not feasible, it should be discussed as an important next step.

3. Results:
Findings: The neural additive model demonstrates superior performance in predicting apnea and hypopnea episodes compared to existing models. Key performance metrics such as accuracy, sensitivity, and specificity are provided, indicating significant improvements.
Interpretation: The discussion effectively relates the results to clinical implications and potential improvements in neonatal care. Additional elaboration on the clinical usability and integration into NICU workflows would be beneficial.

4. Discussion:

Strengths and Limitations: The authors acknowledge some limitations of the study but do not explicitly mention the relatively small sample size or the need for external validation. These aspects should be discussed to provide a more comprehensive view of the study's limitations.

Figures and Tables: The figures and tables are informative and aid in understanding the results. However, table legends should include explanations for any abbreviated terms to ensure they are self-explanatory.

Suggestions for Improvement

1. A thorough comparative analysis with standard models such as logistic regression, support vector machines, or other neural networks was not performed. Including this comparison would provide clearer context for the performance improvements claimed by the authors.

We thank the reviewer for his/her suggestion and agree that a comparison with more traditional baselines would be beneficial.
We have now trained a logistic regression and a multi layer perceptron, which performed the prediction based on a total of 24 engineered features. We computed six features for the higher-frequency oscillatory modalities (NP, T+A, PR): Skewness and kurtosis of the signal in time domain, as well as the centroid, spread, skewness and kurtosis of the power spectrum. For the three lower-frequency modalities (HR, SpO_2, PCO_2), we computed the mean and range (maximum minus minimum value).

With these features, the logistic regression achieved an average AuROC of 0.778, while the multi-layer perceptron achieved an average AuROC of 0.777 (Panel a). These performances are slightly worse than our proposed NAM. We further observed that differences in prediction performance between patients largely persist across these different baselines (Panel b). We have added the relevant figure to the Appendix (Figure A1) and added sections of text in the manuscript (line 166 and line 219), and Appendix (line 525, line 540 and line 547) .

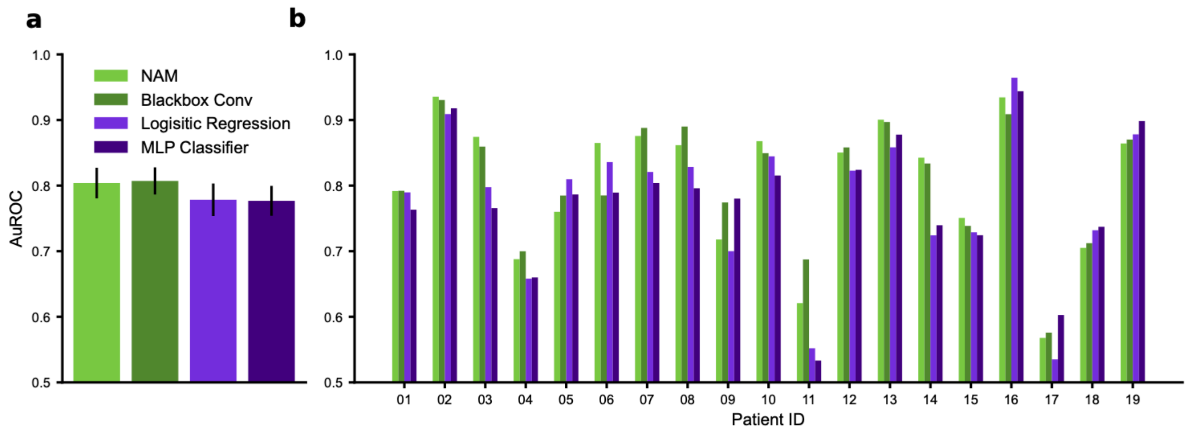

As already mentioned in our manuscript (line 36), the strength of NAM (and deep learning in general) also lies in the fact that no feature engineering is required.

2. While NAMs are inherently interpretable, the manuscript should better address how clinicians can interpret the model's predictions and understand the underlying features contributing to these predictions. Enhancing this aspect could improve the model's acceptance and usability in clinical settings.

Clinicians and NICU personnel are used to polysomnography recordings. As such, the NAM is at an advantage over methods based on (potentially complicated) features or complete blackbox models.
The additive contributions it provides towards decisions are per polysomnography modality. Clinicians can therefore inspect which signal contributed most to the prediction. Furthermore, the activation maps provide a heatmap of important parts in the original signal. As stated previously, this makes them more intuitive to clinicians than non-intuitive features (such as the spectral kurtosis used for our feature-based baselines). We extended the discussion regarding these points in line 306:

Unlike interpretable models based on potentially complicated and unintuitive features (such as for example the spectral moments used in the logistic regression baseline), the explanations provided by the NAM add to the existing polysomnography recording to which practitioners are accustomed: The additive contributions show which of the recorded modalities were relevant to the prediction made, and the activation maps highlight the areas within the recording that were considered discriminative by the model.

3. The study is based on data from one NICU, which may limit the generalizability of the model to other settings with different patient populations or care practices. The manuscript could benefit from discussing strategies for validating the model across diverse clinical environments and patient demographics.

Our current study presents first encouraging results for a patient group with a relatively homogenous pathophysiology of their apneas. We fully agree with the reviewer that applying our prediction model to other patient populations and care practices would be interesting and an important next step, on which we are already working. As suggested, we added a discussion concerning this point to the paper in line 337:

As such, this study represents a first step toward automated systems, but the practical validity of our model requires further study. Because our used dataset consisted of only 19 infants with Robin sequence who have a homogeneous pathophysiology, future studies need to apply the NAM to larger datasets and datasets collected in other settings with different patient demographics and more heterogeneous pathophysiology to provide external validation of our approach.

4. The manuscript provides limited details on the data preprocessing steps and feature selection process. A more comprehensive description of how data was cleaned, how missing values were handled, and how features were selected or engineered would enhance the reproducibility and reliability of the study.

As described in Section 2.3, the only preprocessing we performed was temporal downsampling and standardization/normalization of the seven signal modalities provided by the polysomnography recording. No further feature engineering was performed. We further emphasize the simplicity of our preprocessing in line 122:

No further preprocessing, data cleaning or missing value imputation was performed.

5. There is insufficient discussion on the practical aspects of implementing the neural additive model in real-time clinical settings. Issues such as computational requirements, integration with existing hospital information systems, and real-time data processing need to be addressed to ensure the model can be effectively deployed and used in NICUs.

Related to the point about preprocessing, since we performed very little preprocessing and no feature engineering, preparing time windows for a prediction as well as doing a forward pass through the neural additive model (NAM) does not exhibit any larger computational requirements. We now discuss this in the manuscript in line 316:

The fact that the NAM works directly on minimally pre-processed polysomnography recordings also has computational advantages. In an automated system, the prediction itself could be realized almost instantaneously, leaving enough time to trigger potential interventions. In our study, we consider a prediction window of 15 seconds, which would be enough time to trigger interventions such as sensory stimulation [Lim et al., 2022, Cramer et al. 2018].

6. Although ethical considerations are briefly mentioned, the manuscript does not touch on potential ethical and legal issues related to the use of AI in clinical decision-making. Consider discussing topics such as the need for parental consent, data privacy, and the accountability of AI-driven decisions.

We agree with the reviewer that this needs discussing. We have added the following line to our discussion section (line 343):

Finally, while the use of such automated machine learning systems in neonatal intensive care and health care in general is promising, the use of such systems raises challenging ethical considerations regarding (parental) consent, privacy, and accountability for potential harm.

7. Further discussion on how the authors plan to conduct clinical trials or real-world validation studies would strengthen the credibility and applicability of the findings.

We agree with the reviewer that clinical trials and real-world validation studies are required to investigate the clinical applicability of our model.
Our goal in this study was to provide a proof of principle for predicting individual neonatal apneas and hypopneas in infants with a relatively homogenous pathophysiology and underlying diagnosis (Pierre-Robin sequence) from overnight recordings where no interventions were performed. As such, our set-up is post-hoc, and we extended the discussion about this limitation in line 333:

Furthermore, in this study we were only concerned with performing “post-hoc”' predictions of apneas and hypopneas. In a true interventional setting, the distribution of adverse events is subject to a distribution shift that may affect the performance of our current prediction model.
We also excluded any periods of infant movement from analysis that would need to be dealt with in a real-world application.
As such, this study represents a first step toward automated systems, but the practical validity of our model requires further study. Because our used dataset consisted of only 19 infants with Robin sequence who have a homogeneous pathophysiology, future studies need to apply the NAM to larger datasets and datasets collected in other settings with different patient demographics and more heterogeneous pathophysiology to provide external validation of our approach.

8. The study is based on a relatively small sample of 19 infants. The authors should discuss how this may impact generalizability and whether they conducted power analyses.

We agree with the reviewer that the small sample size is a limitation. We now clearly acknowledge this in the Limitations section (line 338, see paragraph above). We further agree that the important next step is to provide external validation (both with a different cohort of infants with the same pathophysiology, as well as other datasets from different clinical environments or patient demographics). We have extended the Limitations section to incorporate these points in line 340 (see paragraph above).
Nevertheless, we believe that our study presents an important first step, as our NAM model is significantly better than random for 18 out of 19 patients (see the permutation test results in Table A3).

9. More discussion is needed on how the 15-second prediction window translates to clinical utility. Is this sufficient time for intervention?

Since the preprocessing and forward pass to obtain a prediction can be obtained almost instantly, the 15 second time window would be enough time to trigger potential intervening mechanisms like sensory simulation. We have extended the Discussion section in this regard in line 316 (see answer to point 5).

10. Given the clinical setting, a more in-depth analysis of false positives and their potential impact would be beneficial.

The potential impact of false positives depends on the intervention performed. In the most conservative scenario, where the model would not result in an automatic intervention, but only sound an alert to NICU personnel, many false positives could result in alarm fatigue. For more direct interventions via sensory stimulation, the infant could indeed experience more stress than necessary, if the number of false positives is too high. We acknowledge this now in our discussion in line 327. In such cases, having an interpretable model that NICU personnel can distrust as they can more easily inspect why the model is producing the erroneous predictions, is preferable.

A warning system that produces many false negatives would not significantly reduce the stress an infant experiences from repeated apneas and hypopneas. Conversely, a warning system that produces many false positives would potentially place additional stress on the infant by prompting unnecessary interventions.

11. The current approach uses 30-second windows. Did incorporating longer-term dependencies improve prediction?

To answer this question, we performed an experiment, where we doubled the prediction time window from 30 to 60 seconds. Note that, to allow for a fair comparison between both time window durations, it was required to slightly adapt the extraction to ensure that

there is a one-to-one mapping between each 30 second and 60 second time window. Otherwise differences in performance could be due to differences between the datasets used for training and testing the model. See details in the Appendix at line 553.

After ensuring this one-to-one mapping, we found no noticeable difference between the 30 second and 60 second time windows (average AuROC 0.764 vs. 0.762). This is in line with our lag experiment (Figure 1f), which shows that prediction performance degrades substantially with increasing prediction horizon. Clinically, the occurrence of irregularities in the polysomnography recording is expected to happen close to the beginning of an apnea or hypopnea. We added these results to the appendix (line 553) and refer to them in the manuscript in line 215.

12. While interpretability is a strength, it would be interesting to compare performance to "black-box" models to quantify any trade-off.

We agree with the reviewer that it would be interesting to quantify this tradeoff. We have therefore implemented a “blackbox” convolutional neural network as an additional baseline. The architecture is based on the individual single-modality networks, which are used to extract features into a 20 dimensional latent space per feature. The combined 120 dimensional latent space is then passed through a multi-layer perceptron to obtain the final prediction. The model is trained end-to-end on the same time windows and in the same leave-one-out setup used for training the NAM. All details are given together with details on the logistic regression and MLP classifier baseline in the appendix (line 525, line 540 and line 547) .

We find that this blackbox model achieved a similar performance to our more interpretable NAM (average AuROC: 0.807). Like the baselines based on feature engineering (linear regression, MLP classifier), there was no improvement over using the fully non-linear model (Figure A1, see also response to point 1). This suggests that the prediction problem at hand can be solved well by (partly) linear models. As suggested by Rudin (2019), in such cases, interpretable models should be preferred over blackbox models. We have included the results for the blackbox model in the manuscript (line 175 and line 225, and Figure A1).

“Stop explaining black box machine learning models for high stakes decisions and use interpretable models instead”, Rudin, Nature Machine Intelligence, 2019
